# *Tcstv1* and *Tcstv3* elongate telomeres of mouse ES cells

**DOI:** 10.1038/srep19852

**Published:** 2016-01-27

**Authors:** Qian Zhang, Jiameng Dan, Hua Wang, Renpeng Guo, Jian Mao, Haifeng Fu, Xiawei Wei, Lin Liu

**Affiliations:** 1 State Key Laboratory of Medicinal Chemical Biology; 2011 Collaborative Innovation Center for Biotherapy, Department of Cell Biology and Genetics, College of Life Sciences, Nankai University, Tianjin 300071, China; 2 State Key Laboratory of Biotherapy/Collaborative Innovation Center for Biotherapy, West China Hospital, Sichuan University, Chengdu, Sichuan 610041, China

## Abstract

Mouse embryonic stem cell (ESC) cultures exhibit a heterogeneous mixture of metastable cells sporadically entering the 2-cell (2C)-embryo-like state, critical for ESC potency. One of 2-cell genes, *Zscan4,* has been shown to be responsible for telomere maintenance, genomic stability and pluripotency of mouse ESCs. Functions of other 2C-genes in ESCs remain elusive. Here we show that 2C-genes *Tcstv1* and *Tcstv3* play a role in regulation of telomere lengths. Overexpression or knockdown *Tcstv1* and *Tcstv3* does not immediately affect proliferation, pluripotency and differentiation *in vitro* of ESCs. However, ectopic expression of *Tcstv1* or *Tcstv3* results in telomere elongation, whereas *Tcstv1/3* knockdown shortens telomeres of ESCs. Overexpression of *Tcstv1* or *Tcstv3* does not alter telomere stability. Furthermore, *Tcstv1* can increase Zscan4 protein levels and telomere recombination by telomere sister chromatid exchange (T-SCE). Depletion of *Tcstv1/3* reduces Zscan4 protein levels. Together, *Tcstv1* and *Tcstv3* are involved in telomere maintenance that is required for long-term self-renewal of mouse ESCs. Our data also suggests that Tcstv1/3 may co-operate and stabilize Zscan4 protein but the molecular bases remain to be determined.

Mouse ESCs are prototypical pluripotent cells, which are derived from the inner cell mass (ICM) of blastocysts^1^,^2^ and possess similar gene expression patterns compared to ICM cells^3^. They can self-renew and have the capacity to generate tissues of the fetus^4^,^5^. Recently, it has been shown that ESC cultures are a heterogeneous mixture of metastable cells with fluctuating activation of 2-cell embryo specific genes (2C-genes) and endogenous transposable element (TE) activities^6^. These 2C-like cells in ESCs had unique developmental characteristics and could efficiently produce progeny for extraembryonic and embryonic lineages^6^, suggesting that ESCs in the 2C-like state may resemble the totipotent zygotes/2C-stage embryos. Although *Zscan4*, one of the representative 2C-genes^7^,^8^, has been found in restoring and improving the developmental potency of ES/iPS cells^9^,^10^, whether other 2C-genes also function in improving the pluripotency of ESCs is poorly understood.

Mammalian telomeres, containing repetitive G-rich sequences and associated proteins at the ends of linear chromosomes^11^, protect chromosome ends and maintain chromosomal stability^12^,^13^. Telomere length maintenance is primarily regulated by telomerase that adds telomere repeats *de novo* during each cell division, counteracting telomere erosion^14^,^15^,^16^. Recent findings have established that telomeres lengthened rapidly in one- to two-cell stage embryos presumably through telomere recombination or telomere sister chromatid exchange (T-SCE)^17^. Notably, 2C-gene *Zscan4* played important role in lengthening telomeres promptly by recombination-based mechanisms and maintaining genomic stability in ESCs^7^. It remains unclear whether other 2C-genes also play a role in telomere length maintenance, self-renewal and pluripotency of ESCs.

*Tcstv1* (2-cell-stage, variable group, member 1) and *Tcstv3* (2-cell-stage, variable group, member 3) are expressed predominantly in 2-cell embryos^18^,^19^ and transiently in sporadic ESCs^6^,^20^. The two genes share high sequence similarities, but their functions remain largely unknown. Here we show that *Tcstv1* and *Tcstv3* are involved in telomere length maintenance of mouse ESCs.

## Results

### Overexpression of *Tcstv1* or *Tcstv3* does not negatively affect proliferation, pluripotency and differentiation of ESCs

**Figure 1:**
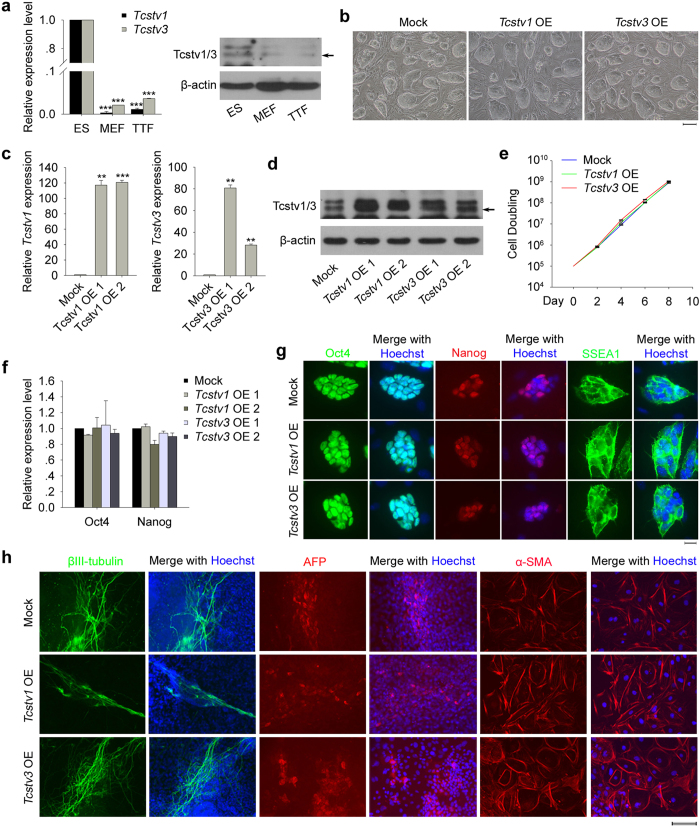
Overexpression of *Tcstv1* or *Tcstv3* does not affect proliferation, pluripotency and differentiation *in vitro* of ESCs. (**a**) *Tcstv1* and *Tcstv3* expression levels in ESCs, MEFs and TTFs by qPCR (two repeated experiments with duplicated samples each) and western blot. (**b**) Morphology of stable *Tcstv1* overexpression, *Tcstv3* overexpression and mock ESCs. Scale bar = 100 μm. (**c**) Confirmation of overexpression of *Tcstv1* and *Tcstv3* in respective OE ESCs by qPCR. (**d**) Western blot experiment confirmed increased expression of Tcstv1 and Tcstv3 protein (pointed by black arrow). (**e**) Growth curves of *Tcstv1* OE, *Tcstv3* OE and mock ESCs. 1 × 10^5^ cells were seeded on feeder cells on Day 0 and cells were counted and passaged every two days. (**f**) Expression levels of *Oct4* and *Nanog* showed no obvious differences among *Tcstv1* OE, *Tcstv3* OE and mock ESCs by qPCR analysis. (**g**) Ectopic expression of *Tcstv1* or *Tcstv3* did not change expression of pluripotency-associated genes by fluorescence microscopy. Scale bar = 20 μm. (**h**) Overexpression of *Tcstv1* or *Tcstv3* did not affect differentiation capacity *in vitro* of ESCs by immunofluorescence staining of markers for ectoderm (βIII-tubulin), endoderm (AFP) and mesoderm (α-SMA). Scale bar = 100 μm. **P < 0.01, ***P < 0.001, compared to controls.

We confirmed that *Tcstv1* and *Tcstv3* were highly expressed in ESCs, while their expression levels were low in mouse embryonic fibroblasts (MEFs) and tail-tip fibroblasts (TTFs) (Fig. 1a), implying that *Tcstv1/3* may play important roles in ESCs. To understand the role of *Tcstv1/3* in ESCs, we generated *Tcstv1* and *Tcstv3* respectively stable ectopic expression/overexpression (OE) ESCs using established naive ESC lines^21^. Morphologically, *Tcstv1* OE and *Tcstv3* OE ESCs showed compact cell colonies like mock ESCs transfection with empty vector (Fig. 1b). Increased expression levels of *Tcstv1* and *Tcstv3* in their respective OE ESCs were confirmed by quantitative real-time PCR (qPCR; Fig. 1c). We generated a polyclonal antibody against both Tcstv1 and Tcstv3 protein due to their quite similar amino acids sequence, with two bands closely related. By western blot, we confirmed noticeable Tcstv1 protein overexpression in *Tcstv1* OE ESCs and Tcstv3 protein overexpression in *Tcstv3* OE ESCs (Fig. 1d).

Cell proliferation did not differ in *Tcstv1* OE, *Tcstv3* OE and mock ESCs by culture over four passages (Fig. 1e). Furthermore, ectopic expression of *Tcstv1* or *Tcstv3* did not alter expression of pluripotency-associated genes by qPCR analysis (Fig. 1f) and immunofluorescence (Fig. 1g). To test whether *Tsctv1* and *Tcstv3* play a role in differentiation of ESCs, we differentiated *Tcstv1* OE, *Tcstv3* OE and mock ESCs *in vitro* by embryoid body (EB) formation. Markers for three germ layers, βIII-tubulin (ectoderm), alpha 1-fetoprotein (AFP, endoderm) and alpha smooth muscle actin (α-SMA, mesoderm) were expressed similarly on day 15 (Fig. 1h). These data indicate that ectopic expression of *Tcstv1* or *Tcstv3* does not immediately affect proliferation, pluripotency and differentiation *in vitro* of mouse ESCs.

### *Tcstv1* and *Tcstv3* elongate telomere lengths in mouse ESCs

**Figure 2:**
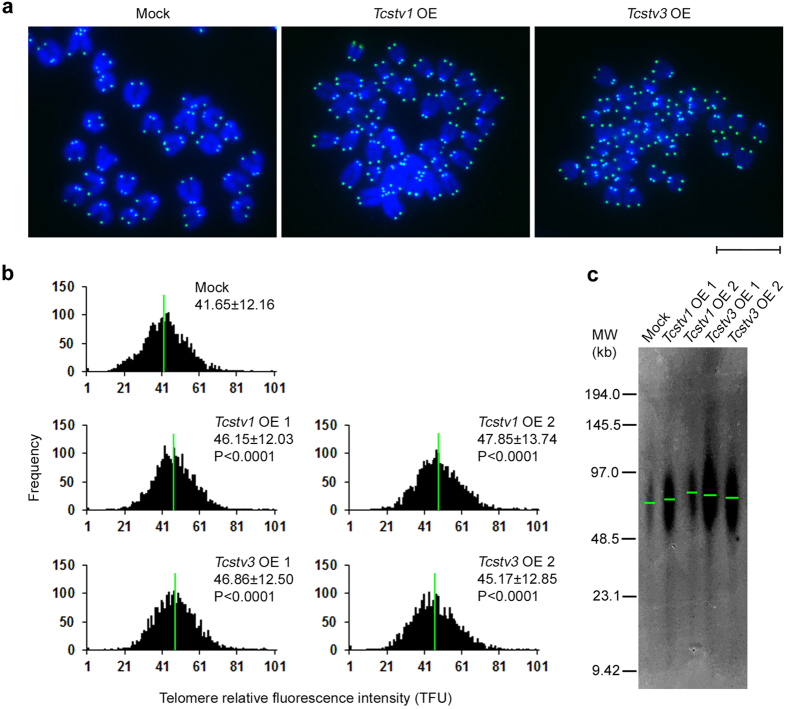
*Tcstv1* and *Tcstv3* extend telomere lengths in mouse ESCs. (**a**) Representative telomere Q-FISH images of *Tcstv1* OE, *Tcstv3* OE and mock ESCs at P10. Telomeres are labeled with telomere PNA probes (green), and chromosomes are labeled with DAPI (blue). Scale bar = 10 μm. (**b**) Histogram shows distribution of relative telomere length expressed as fluorescence intensity (TFU, telomere fluorescence unit) by telomere Q-FISH analysis. Green line is median telomere length. Average telomere length is shown as mean TFU ± SD. P value, compared to mock ESCs. (**c**) Telomere restriction fragment (TRF) analysis showed increased telomere lengths in *Tcstv1* OE and *Tcstv3* OE ESCs compared with mock ESCs at P9.

*Tcstv1* and *Tcstv3* are specific genes for mouse ESCs and the 2-cell embryos. We tested whether they function in regulation of telomere lengths in ESCs, like *Zscan4*. We measured telomere lengths of *Tcstv1* OE, *Tcstv3* OE and mock ESCs by telomere quantitative fluorescence *in situ* hybridization (Q-FISH) analysis^22^, following culture for 10 passages (Fig. 2a). Relative telomere lengths shown as telomere fluorescence intensity (TFU) were significantly (P < 0.0001) longer in *Tcstv1* OE ESCs (46.15 ± 12.03 TFU in OE 1 and 47.85 ± 13.74 TFU in OE 2 ESCs) and *Tcstv3* OE ESCs (46.86 ± 12.50 TFU in OE 1 and 45.17 ± 12.85 TFU in OE 2 ESCs), compared to mock ESCs (41.65 ± 12.16 TFU; Fig. 2b). To validate the results by Q-FISH analysis, we also measured telomere lengths using Southern blot-based terminal restriction fragment (TRF) analysis^23^ at P9. Consistent with the Q-FISH data, telomeres were elongated in *Tcstv1* OE and *Tcstv3* OE ESCs compared with mock ESCs (Fig. 2c). Furthermore, we measured telomere lengths by Q-FISH analysis of ESCs following culture for additional passages (at P15). Again, telomeres were longer in *Tcstv1* OE and *Tcstv3* OE ESCs than in mock ESCs (Supplementary Fig. 1a), consistent with quantitative real-time PCR data shown as T/S ratio^24^ (Supplementary Fig. 1b).

We also established stable *Tcstv1* OE and *Tcstv3* OE ESCs using feeder-free J1 ESC lines, and confirmed increased expression levels of *Tcstv1* and *Tcstv3* by quantitative real-time PCR and western blot (Supplementary Fig. 2a, 2b). By Q-FISH analysis at P7, telomeres were significantly (P < 0.0001) lengthened in *Tcstv1* OE ESCs compared to both J1 mock-1 and mock-2 ESCs, and telomeres of *Tcstv3* OE ESCs significantly (P < 0.0001) lengthened compared to that of J1 mock-1 ESCs (Supplementary Fig. 2c). These data indicate that *Tcstv1* and *Tcstv3* can promote telomere elongation of mouse ESCs.

To test whether *Tcstv1* and *Tcstv3* can maintain telomere stability and reduce DNA damage at telomeres in ESCs, we performed immunofluorescence analysis (Supplementary Fig. 3a). Co-localization of γH2AX and TRF1 foci (TIFs), indicative of telomere-induced DNA damage^25^,^26^, showed no significant difference between *Tcstv1* OE or *Tcstv3* OE ESCs and mock ESCs (Supplementary Fig. 3b), suggesting that *Tcstv1* or *Tcstv3* overexpression does not influence telomere stability of ESCs.

### *Tcstv1* and *Tcstv3* enhance slightly telomere sister chromatid exchange (T-SCE) in mouse ESCs partly dependent of Zscan4

**Figure 3:**
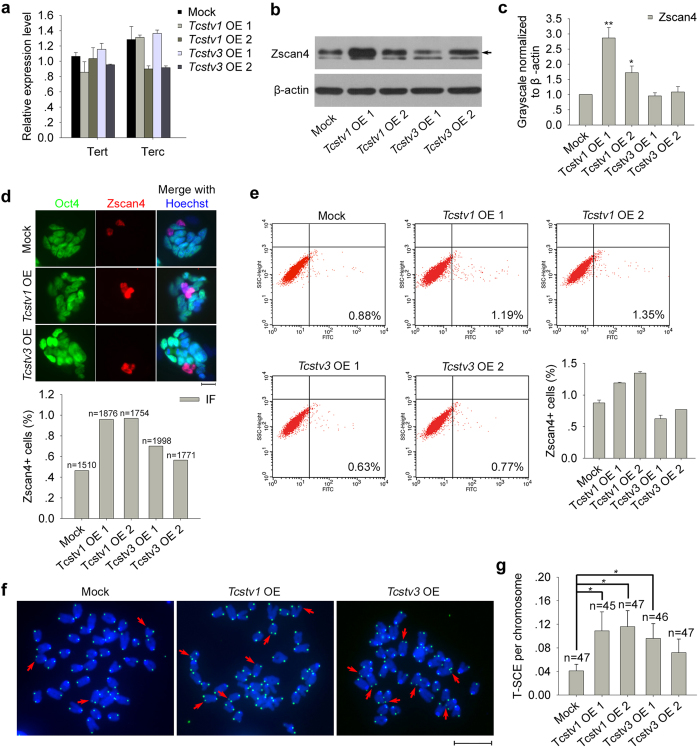
*Tcstv1* or *Tcstv3* overexpression enhances telomere sister chromatid exchange (T-SCE) in mouse ESCs and elevates Zscan4 protein levels. (**a**) Expression levels of *Tert* and *Terc* showed no significant difference among *Tcstv1* OE, *Tcstv3* OE and mock ESCs by qPCR analysis. Two repeated experiments with duplicated samples each. (**b**) Confirmation of Zscan4 protein levels in *Tcstv1* OE, *Tcstv3* OE and mock ESCs by western blot. Black arrow indicates Zscan4 bands. (**c**) Quantification of relative Zscan4 protein levels normalized to β-actin by ImageJ software. n = 4. (**d**) Immunofluorescence images of Zscan4 (red) and Oct4 (green) in *Tcstv1* OE, *Tcstv3* OE and mock ESCs (Scale bar = 20 μm), and proportion of Zscan4 positive cells. n, number of cells counted. χ^2^ test shows no statistical difference (P > 0.05). (**e**) Analysis of Zscan4^+^ cells in *Tcstv1* OE, *Tcstv3* OE and mock ESCs by flow cytometry. (**f**) Representative micrographs showing T-SCE (red arrows) by CO-FISH analysis. Scale bar = 10 μm. (**g**) T-SCE per chromosome in *Tcstv1* OE, *Tcstv3* OE and mock ESCs. n, number of cells counted. *P < 0.05, **P < 0.01, compared with controls.

To understand the mechanisms underlying *Tcstv1* and *Tcstv3* functions in telomere elongation of ESCs, we first considered the telomerase, primary enzyme responsible for telomere maintenance. Expression of telomerase subunit *Tert* and *Terc* remained at similar levels among *Tcstv1* OE, *Tcstv3* OE and mock ESCs (Fig. 3a), suggesting that *Tcstv1* or *Tcstv3* overexpression does not significantly increase telomerase activity. Perhaps telomerase independent mechanism is activated in *Tcstv1/3* OE ESCs.

Since *Zscan4* is responsible for telomere lengthening of ESCs independent of telomerase, we wondered whether overexpression of *Tcstv1* or *Tcstv3* can increase expression of *Zscan4*. The protein levels of Zscan4, by statistics for four independent western blot experiments, increased significantly in *Tcstv1* OE ESCs compared with mock ESCs (Fig. 3b,c). By immunofluorescence microscopy, Zscan4 was expressed sporadically in only small proportion of ESC cultures (Fig. 3d), consistent with previous reports^6^,^7^. Furthermore, proportion of Zscan4 positive cells was increased in *Tcstv1* OE ESCs by both immunofluorescence microscopy quantification and flow cytometry analysis (Fig. 3d,e). We also measured genes expressed in 2C-like state of ESCs, including *Zscan4c* (predominant transcript of *Zscan4* gene cluster in ESCs^8^), *Dub1*, *Dazl*, *Ott* and *Eif1a-like*^27^,^28^, by qPCR analysis (Supplementary Fig. 4a). Despite increased expression in *Tcstv1* OE 1 ESCs, overall their expression levels in *Tcstv1* OE and *Tcstv3* OE ESCs remained not much change, compared with mock ESCs. Retrotransposons, expressed when the zygotic genome is first transcribed, including murine endogenous retrovirus with leucine tRNA primer (*MuERV-L*), long interspersed nuclear element-1 (*LINE-1*), and the non-autonomous short interspersed elements (*SINEs*)^6^,^29^, were expressed at similar levels among *Tcstv1* OE*, Tcstv3* OE and mock ESCs (Supplementary Fig. 4b).

Histone modifications and repressive DNA methylation at telomeres and subtelomeres are important regulators of mammalian telomere lengths^30^. We measured related histone and DNA methylation levels by western blot. Active histones H3K9Ac, H3Ac and heterochromatic repressive H3K9me3 did not differ in their protein levels between *Tcstv1* OE and mock ESCs (Supplementary Fig. 5a). The protein levels of DNA methyltransferases Dnmt3a and Dnmt3b which can methylate hemimethylated and unmethylated DNA, showed no significant differences either (Supplementary Fig. 5b). Despite whole protein levels, we also performed ChIP-qPCR using anti-Dnmt3b to analyze binding of Dnmt3b to subtelomeres following *Tcstv1* overexpression. As expected, mock ESCs served as control exhibited enrichment of Dnmt3b at subtelomeres of chromosomes 7 and 13. However, *Tcstv1* OE and mock ESCs showed similar Dnmt3b enrichment at subtelomeres (Supplementary Fig. 5c).

Moreover, we tested whether *Tcstv1* and *Tcstv3* influence telomere sister chromatid exchange (T-SCE) in ESCs by a telomere chromosome orientation FISH (CO-FISH) assay^31^,^32^ (Fig. 3f). Frequency of T-SCE was increased in *Tcstv1* OE and *Tcstv3* OE 1 ESCs compared to that of mock ESCs (Fig. 3g). Considering that *Zscan4* expressed at higher levels in *Tcstv1* OE ESCs, we speculated that *Tcstv1* and *Tcstv3* may enhance T-SCE efficiency by increasing Zscan4 levels to elongate telomeres in ESCs. However, the frequency of T-SCE (Fig. 3g) did not completely correlate with Zscan4 protein levels (Fig. 3c), suggesting that factors other than Zscan4 may also play roles in T-SCE and telomere elongation of *Tcstv1* OE and *Tcstv3* OE ESCs.

### *Tcstv1/3* knockdown does not affect proliferation, pluripotency and differentiation *in vitro* of ESCs

**Figure 4:**
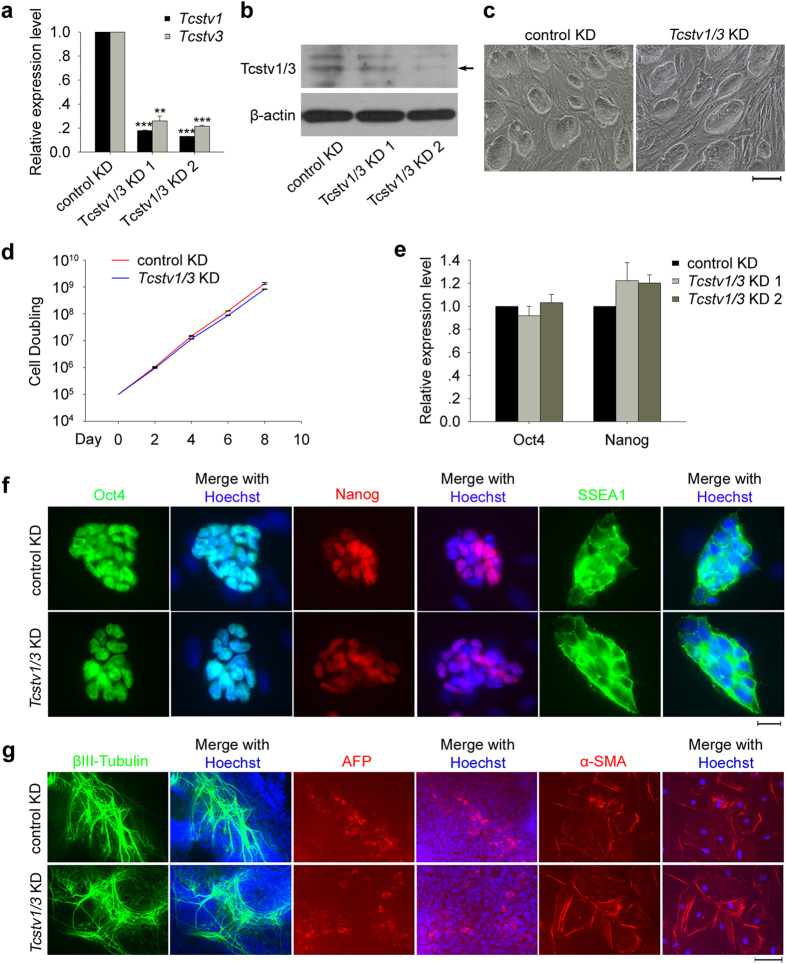
*Tcstv1*/*3* knockdown does not influence proliferation, pluripotency and differentiation *in vitro* of ESCs. (**a**) Reduced expression of *Tcstv1* and *Tcstv3* was confirmed by qPCR analysis in stable KD ESCs generated by shRNA1 construct. **P < 0.01, ***P < 0.001, compared to controls. (**b**) Confirmation of Tcstv1 and Tcstv3 protein (pointed by black arrow) decreased expression in KD ESCs by western blot. (**c**) Morphology of *Tcstv1/3* stable KD ESCs and control KD ESCs. Scale bar = 100 μm. (**d**) Growth curves of *Tcstv1/3* KD and control KD ESCs. 1 × 10^5^ cells were seeded on feeder cells on Day 0 and cells were counted and passaged every two days. n = 3. (**e**) *Oct4* and *Nanog* expression levels showed no significant difference between *Tcstv1/3* KD and control KD ESCs by qPCR analysis. (**f**) *Tcstv1/3* knockdown did not alter expression of pluripotency-associated genes by fluorescence microscopy. Scale bar = 20 μm. (**g**) *Tcstv1/3* KD did not influence differentiation capacity *in vitro* of ESCs by immunofluorescence staining of three germ layer markers, βIII-tubulin (ectoderm), AFP (endoderm) and α-SMA (mesoderm). Scale bar = 100 μm.

To validate the findings obtained by overexpression of *Tcstv1* or *Tcstv3*, we established *Tcstv1/3* knockdown (KD) ESCs by RNA interference using two shRNA constructs. The mRNA sequences of *Tcstv1* and *Tcstv3* are quite similar to each other, so that we hardly designed shRNA targeting each one respectively. Two shRNA constructs can both target *Tcstv1*, and shRNA1 can also target *Tcstv3* and other five genes with unknown functions (Table S1), because of their high similar mRNA sequences with *Tcstv1* and *Tcstv3*. The mRNA expression levels of *Tcstv1* and *Tcstv3* in two KD ESCs generated by shRNA1 were both effectively reduced to about 20% of that of control KD ESCs at P8 (Fig. 4a). However shRNA2 could not decrease *Tcstv1* mRNA expression level effectively (data not shown), thus the two stable KD ESC lines used for the following experiments were generated by shRNA1 if not otherwise mentioned. Western blot experiments confirmed reduced protein levels of *Tcstv1* and *Tcstv3* in KD ESCs (Fig. 4b).

*Tcstv1/3* knockdown ESCs maintained characteristics of ESCs in morphology, like control KD ESCs, displaying large nuclei and nucleoli under higher magnification with clear compact clonal boundaries (Fig. 4c). *Tcstv1/3* KD did not impact ESC proliferation (Fig. 4d), nor expression of common ESC marker genes by qPCR analysis (Fig. 4e) and by immunofluorescence (Fig. 4f). Furthermore, *Tcstv1/3* knockdown did not reduce differentiation of ESCs to three embryonic germ layers *in vitro* by standard embryoid body formation test (Fig. 4g).

### *Tcstv1/3* knockdown shortens telomeres in mouse ESCs and decreases *Zscan4* expression

**Figure 5:**
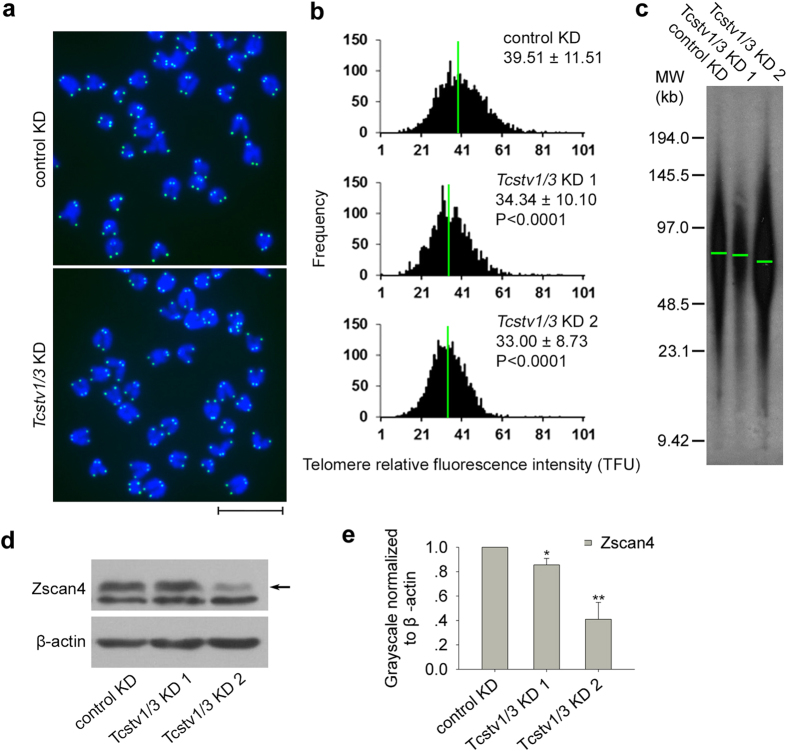
*Tcstv1/3* knockdown reduces telomere lengths in mouse ESCs and decreases Zscan4 protein levels. (**a**) Representative telomere Q-FISH images of *Tcstv1/3* KD and control KD ESCs at P8. Telomeres are labeled with telomere PNA probes (green), and chromosomes are labeled with DAPI (blue). Scale bar = 10 μm. (**b**) Histogram shows distribution of relative telomere length expressed as TFU (telomere fluorescence unit) by telomere Q-FISH analysis. Average telomere length is shown as mean TFU ± SD. P value, compared to control KD ESCs by t-test. (**c**) Telomere restriction fragment (TRF) analysis showed decreased telomere lengths in *Tcstv1/3* KD ESCs compared with control KD ESCs at P8. (**d**) Confirmation of Zscan4 expression levels in *Tcstv1/3* KD and control ESCs by western blot. Zscan4 band is indicated by black arrow. (**e**) Relative Zscan4 protein quantity normalized to β-actin by ImageJ software. n = 4; *P < 0.05, **P < 0.01, compared to controls.

Q-FISH analysis was performed in N33 *Tcstv1/3* KD ESCs and control ESCs at P8 to measure telomere lengths (Fig. 5a). Telomeres were shorter in *Tcstv1/3* KD ESCs (34.34 ± 10.10 TFU in KD 1 and 33.00 ± 8.73 TFU in KD 2 ESCs) than control ESCs (39.51 ± 11.51 TFU), with significant difference (P < 0.0001; Fig. 5b). Consistently, TRF analysis at P8 showed shorter telomeres in *Tcstv1/3* KD ESCs compared with control ESCs (Fig. 5c).

We also established *Tcstv1/3* stable knockdown ESCs by shRNA1 using F1 ESC lines, and confirmed reduced expression levels of *Tcstv1* and *Tcstv3* by quantitative real-time PCR (Supplementary Fig. 6a). By Q-FISH analysis at P8, telomeres were shorter (P < 0.0001) in *Tcstv1/3* KD ESCs than in F1 control KD ESCs (Supplementary Fig. 6b), further supporting the notion that *Tcstv1* and *Tcstv3* function in telomere length maintenance of mouse ESCs.

Also, Zscan4 protein expression levels declined in *Tcstv1/3* KD ESCs by western blot experiments (Fig. 5d,e). However, genes expressed in 2C-like state of ESCs and retrotransposons showed no significant reduction or only minimal changes in *Tcstv1/3* KD ESCs by qPCR analysis (Supplementary Fig. 7a, 7b). Furthermore, *Tcstv1/3* knockdown did not alter histone modification levels (Supplementary Fig. 8a), expression of Dnmt3a and Dnmt3b (Supplementary Fig. 8b), and binding of Dnmt3b at subtelomeres (Supplementary Fig. 8c). These data suggest that *Tcstv1/3* knockdown decreases Zscan4 protein levels and shortens telomeres.

## Discussion

Here we show that *Tcstv1* and *Tcstv3* can extend telomere lengths through telomere sister chromatid exchange (T-SCE) partly dependent of Zscan4. *Tcstv1* and *Tcstv3* share high sequence and function similarities, and locate nearby at subtelomeric regions on chromosome 13^33^, predicted to be in the same gene cluster. The genes targeted by shRNA1 construct (Table S1), including *AF067061*, *BC147527*, *Gm20767*, *B020031M17Rik* and *Gm21818*, located nearby and sharing high similar sequences with *Tcstv1* and *Tcstv3*, may be also included in the same gene cluster and have similar functions. Knockdown by shRNA1 in ESCs also may alter expression of the gene cluster at low levels, not just *Tcstv1* and *Tcstv3*.

*Tcstv1*, *Tcstv3* and *Zscan4* all locate at subtelomeric regions and share the same upstream genes and mechanisms^6^,^27^,^33^,^34^,^35^. Previous report showed that transiently over-expressed *Tcstv1* and *Tcstv3* did not change *Zscan4* mRNA expression levels^27^, and confirmed here, suggesting that *Tsctv1* and *Tcstv3* may not directly regulate *Zscan4*. Here we observed increased protein levels of *Zscan4* in *Tcstv1* OE ESCs and decreased Zscan4 protein levels in *Tcstv1/3* KD ESCs. We speculated that *Tsctv1/3* may affect Zscan4 protein stability. Another possibility is that Tcstv1, Tcstv3 and Zscan4 may function synergistically in T-SCE, since T-SCE frequency also is increased in *Tcstv3* OE ESCs without elevating Zscan4. Furthermore, higher Zscan4 expression levels and more frequency of T-SCE in *Tcstv1* OE than *Tcstv3* OE ESCs suggest that *Tcstv1* may play more roles in regulating Zscan4 expression and telomere length maintenance than does *Tcstv3*. How *Tcstv1/3* regulates *Zscan4* remains interesting in future studies.

Telomere lengths are associated with authentic pluripotency of ES/iPS cells. In spite of no obvious changes in expression of most pluripotency-associated genes, ESCs with short telomeres show decreased proliferative rate, reduced teratoma formation and chimera production, and fail to generate complete ESC pups^21^. We show that *Tcstv1* and *Tcstv3* overexpression or knockdown influences telomere lengths of mouse ESCs following 8–15 passages, despite to less extent compared with role of *Zscan4* itself 7. Telomeres are long in mice compared to humans and particularly long in mouse ESCs^21^,^23^,^36^. It is not surprising that the slow changes in telomere lengths by *Tcstv1* or *Tcstv3* overexpression or knockdown do not immediately impact proliferation and differentiation *in vitro* of ESCs. We recognize the limitation of knockdown experiments using RNAi technology here. It will be interesting in the future to test the effect of complete depletion of *Tcstv1/3* in ESCs by knockout of *Tcstv1/3* using new technology CRISPR/Cas9. Moreover, attempts in generating *Tcstv1* and *Tcstv3* knockout mice will answer questions of whether *Tcstv1* and *Tcstv3* are required for developmental pluripotency *in vivo.*

## Methods

### Mouse ESCs

N33 ESC lines were derived from C57BL/6 mice^21^, and F1 ESC lines were derived from B6C3F1 mice^37^. J1 ESCs were cultured without feeder. The ESC culture medium consisted of knock-out DMEM (Invitrogen) with 20% FBS (Hyclone), 1000 U/ml mouse leukemia inhibitory factor (LIF; ESG1107; Millipore), 0.1 mM non-essential amino acids, 0.1 mM β-mercaptoethanol, 1 mM L-glutamine, penicillin (100 U/ml) and streptomycin (100 μg/ml). For culture of ESCs, the medium was changed daily, and cells were routinely passaged every two days.

### Generation of *Tcstv1* OE, *Tcstv3* OE and stable *Tcstv1/3* knockdown ESCs

Murine *Tcstv1* and *Tcstv3* CDS were cloned into expression vector pCAGIpuro (pLch37) at XhoI/NotI sites. N33 (at P13) and J1 ESCs were transfected with 2 μg linearized pCAGIpuro-*Tcstv1*, pCAGIpuro-*Tcstv3* vector or empty vector served as control using lipofectamine TM2000 (Invitrogen) and then selected with 2 μg/ml puromycin for about one week. The resistant clones were picked to achieve stable *Tcstv1* overexpression, *Tcstv3* overexpression or mock ESC lines.

Control and two different shRNA sequences against *Tcstv1/3* mRNA were used for *Tcstv1/3* knockdown experiments. The sequences were cloned into pSIREN-RetroQ (Clontech) and the resultant vectors were introduced into Plat-E cells to package retrovirus. N33 (at P13) and F1 (at P17) ESCs were then infected with control and *Tcstv1/3* RNAi retrovirus, and selected with 2 μg/ml puromycin for about one week. The resistant clones were picked. The 19 nuclotide sequences of *Tcstv1/3* shRNA are listed in Table S2. Since shRNA2 could not decrease *Tcstv1* expression effectively, two knockdown ESC lines generated by shRNA1 and control KD ESC lines generated by control shRNA were used for the following experiments if not otherwise mentioned.

### Embryoid body formation test

Embryoid body (EB) formation *in vitro* was performed as described previously^38^. ESCs were removed off feeder cells twice based on their differences in the adherence to the bottom of dish. The cells were diluted to 4 × 10^4^ per milliliter. Every 30 μL was pipetted to form a hanging drop on the cover of the 100-mm dish. Embryoid bodies (EBs) formed on day 4, and then were transferred to six-well plates for adherent culture. EBs were fixed for immunofluorescence staining using markers of three embryonic germ layers on day 15.

### Gene expression analysis by quantitative real-time PCR

Total RNA was purified using a RNA mini kit (Qiagen), treated with DNase I (Qiagen), and the cDNA was generated from 2 μg RNA using Oligo(dT) 18 primer (Takara) and M-MLV Reverse Transcriptase (Invitrogen). Real-time quantitative PCR reactions were set up in duplicate with the FS Universal SYBR Green Master (Roche) and carried out on an iCycler MyiQ2 Detection System (BIO-RAD). All reactions were carried out by amplifying target genes and internal control in the same plate. Each sample was repeated 2 or 3 times and normalized using GAPDH as the internal control. The amplification was performed for primary denaturation at 95 °C for 10 min, then 40 cycles of denaturation at 95 °C for 15 s, annealing and elongation at 58 °C for 1 min, and the last cycle under 55–95 °C for dissociation curve. Relative quantitative evaluation of target gene was determined by comparing the threshold cycles. Primers were confirmed their specificity with dissociation curves. Most primers were designed using the IDT DNA website and primers used are listed in Table S3.

### Western blot

Cells were washed twice in PBS, collected, and lysed in cell lysis buffer on ice for 30 min and then sonicated for 1 min at 60 of amplitude with 2 s intervals. After centrifugation at 10,000g, 4 °C for 10 min, supernatant was transferred into new tubes. The concentration of the protein sample was measured by bicinchoninic acid, and then protein samples were boiled in SDS Sample Buffer at 99 °C for 10 min. 20 μg or 40 μg (for Tcstv1/3 and Zscan4) total proteins of each cell extracts were resolved by 10% or 12% (for Tcstv1/3) Bis-Tris SDS-PAGE and transferred to polyvinylidene difluoride membranes (PVDF; Millipore). Nonspecific binding was blocked by incubation in 5% skim milk in TBST at room temperature for 2 h. Blots were then probed with primary antibodies, Tcstv1/3 (custom-made), Zscan4 (AB4340; Millipore), H3K9me3(ab8898; Abcam), H3K9Ac (04-1003; Millipore), H3Ac (06-599; Millipore), H3 (ab1791; Abcam), Dnmt3a (ab13888; Abcam), Dnmt3b (ab13604; Abcam) and β-actin (P30002; Abmart) by overnight incubation at 4 °C in 5% skim milk in TBST. Immunoreactive bands were then probed for 2 h at room temperature with the appropriate horseradish peroxidase (HRP)-conjugated secondary antibodies, anti-Rabbit IgG-HRP (GE Healthcare, NA934V), or goat anti-Mouse IgG (H + L)/HRP (ZSGB-BIO, ZB-2305). Protein bands were detected by Chemiluminescent HRP substrate (Millipore, WBKLS0500).

### Immunofluorescence microscopy

Cells were washed twice in PBS, then fixed in freshly prepared 3.7% paraformaldehyde in PBS (pH 7.4) for 30 min at 4 °C, washed in PBS for one time and permeabilized in 0.1% Triton X-100 in blocking solution (3% goat serum plus 0.1% BSA in PBS) for 30 min at room temperature, then washed in PBS for one time, and left in blocking solution for 2 h. Cells were incubated overnight at 4 °C with primary antibodies against Oct4 (sc5279; Santa Cruz), Nanog (ab80892; Abcam), SSEA-1 (MAB4301; Millipore), βIII-tubulin (CBL412; Chemicon), alpha 1-fetoprotein (AFP; DAK-N1501; Dako), alpha smooth muscle actin (α-SMA; ab5694-100; Abcam), γH2AX (05-636; Millipore), TRF1 (TRF12-S; Alpha Diagnostic) and Zscan4 (AB4340; Millipore). Then cells were washed three times (each for 15 min) with blocking solution, and incubated for 2 h with secondary antibodies at room temperature. Goat Anti-Mouse IgG (H + L) FITC (115-095-003; Jackson) and Goat Anti-Rabbit IgG (H + L) Alexa Fluor^®^ 594 (111-585-003; Jackson), diluted 1:200 with blocking solution, were used. Samples were washed, and counterstained with 0.5 μg/ml Hoechst 33342 (H1398; MP) in Vectashield mounting medium. Fluorescence was detected and imaged using a Zeiss Axio-Imager Z1 fluorescence microscope.

### Flow cytometry analysis

ESCs were collected and washed in cold PBS, then fixed in cold 70% ethanol. Cells were permeabilized in 0.1% Triton X-100 in blocking solution (3% goat serum in PBS) for 30 min, then washed and left in blocking solution for 1 h. ESCs were incubated with primary antibodies against Zscan4 (AB4340; Millipore), washed three times, and incubated for 1 h with secondary antibodies, 488 goat anti-rabbit IgG (A11008; Invitrogen). Samples were washed three times with PBS and fluorescence activated cell sorting (FACS) analysis was performed using a Flow Cytometer (BD FACS Calibur).

### Telomere quantitative fluorescence *in situ* hybridization (Q-FISH)

Telomere length and function (telomere integrity and chromosome stability) was estimated by Q-FISH as described previously^39^,^40^. Cells were incubated with 0.3 μg/ml nocodazole for 3h to enrich cells at metaphases. Chromosome spreads were made by a routine method. Metaphase-enriched cells were exposed to hypotonic treatment with 0.075 M KCl solution, fixed with methanol: glacial acetic acid (3:1) and spread onto clean slides. Telomeres were denatured at 80 °C for 3 min and hybridized with FITC-labeled (CCCTAA)_3_ peptide nucleic acid (PNA) probe at 0.5 μg/ml (Panagene, Korea). Chromosomes were counter-stained with 0.5 μg/ml DAPI. Fluorescence from chromosomes and telomeres was digitally imaged on a Zeiss Imager Z2 microscope with FITC/DAPI filters, using AxioCam and AxioVision software 4.6. For quantitative measurement of telomere length, telomere fluorescence intensity was integrated using the TFL-TELO program (a gift kindly provided by P. Lansdorp, Terry Fox Laboratory), and calibrated using standard fluorescence beads.

### Telomere restriction fragment (TRF) analysis

The TRF analysis was performed using a commercial kit (TeloTAGGG Telomere Length Assay, catalog no. 12209136001, Roche Life Science), based on the method described previously^23^ with slight modifications. Cells were isolated and embedded in agarose plugs (Pulsed Field Certified Agarose, 162-0137, Bio-Rad) to let plugs containing 5 × 10^5^ cells and treated with Proteinase K (PCR Grade, 03115879001, Roche Life Science). Then the plug was digested with MboI (R0147L, NEB) for 15 h and underwent electrophoresis through a 1% agarose gel in 1 × TAE at 14 °C for 16 h at 6 V/cm with an initial pulse time of 1 s and end in 12 s using the Bio-Rad CHEF DR-III pulse-field system. The gel was blotted and probed using reagents in the kit.

### Telomere measurement by quantitative real-time PCR

Quantitative real-time PCR (qPCR) was used to measure relative telomere lengths (RTL) as previously described^24^. Genome DNA was extracted from cells using DNeasy Blood & Tissue Kit (Qiagen). Average telomere length was measured from total genomic DNA using a real-time PCR assay, modified for measurement of mouse telomeres. For each sample, 20 ng of genome DNA was used in each reaction. PCR reactions were performed on an iCycler MyiQ2 Detection System (BIO-RAD), using telomeric primers and primers for the reference control gene (mouse 36B4 single-copy gene)^24^ (Table S4). For each PCR reaction, a standard curve was made by serial dilutions of known amounts of mouse genomic DNA. The telomere signal was normalized to the signal from the single-copy gene to generate a T/S ratio indicative of relative telomere length.

### ChIP-qPCR analysis

ChIP-qPCR analysis was performed as described previously^27^. Briefly, cells were harvested and fixed by freshly prepared 1% paraformaldehyde solution for 10 min at room temperature. Their nuclei extracted, lysed, and sonicated. DNA fragments were then enriched by immunoprecipitation with 6 μg Dnmt3b antibody (ab13604; Abcam). The eluted protein:DNA complex was reverse-crosslinked at 65 °C overnight. DNA was recovered after proteinase and RNase A treatment. ChIP-enriched DNA was analyzed by qPCR using primers for subtelomeres (Table S5). Mouse (G3A1) mAb IgG1 Isotype Control (Cell Signaling, 5415S) served as negative control.

### Telomere Chromatid Orientation-Fluorescence *In Situ* Hybridization (CO-FISH)

CO-FISH assay was performed as described previously^41^, with minor modification. Subconfluent cells were incubated with BrdU (10 μM) for 10–12 h. Nocodazole with 0.3 μg/ml was added for 3h prior to cell harvest, and metaphase spreads were prepared by a routine method. Chromosome slides were treated with RNase A, fixed with 4% formaldehyde, then stained with Hoechst 33258 (0.5 mg/ml), incubated in 2 × SSC (Invitrogen) for 15 min and exposed to 365 nm UV light (Stratalinker 1800UV irradiator) for 40 min. The BrdU-substituted DNA was digested with Exonuclease III (Takara). The slides were then dehydrated through ethanol series and air dried. PNA-FISH was performed with FITC-OO-(CCCTAA)_3_ (Panagene, F1009). Slides were hybridized, washed, dehydrated, mounted, and counter-stained with 1.25 μg/ml DAPI in VectaShield antifade medium. Digital images were captured using a CCD camera on a Zeiss Imager Z2 microscope.

### Generation of Tcstv1/3 antibodies

Custom-made polyclonal rabbit anti-Tcstv1/3 antibodies were generated (Genscript) against the epitope of Tcstv1: CQRKPKVSPGDVENY (the C-terminal cysteine was added for KLH conjugation). This peptide is exactly matched to predicted amino acids sequence of Tcstv1 protein and quite similar to Tcstv3 protein (QR**E**P**Q**VSPGDVENY, different amino acids in bold), thus the antibody can recognize both Tcstv1 and Tcstv3 proteins.

### Statistical analysis

All results were analyzed by student’s t-test or χ^2^ test (specially mentioned) and the resulting P-values were shown. Significant differences were defined as *P < 0.05, **P < 0.01, or ***P < 0.001. The results were shown as mean ± SEM unless other instructions were indicated.

## Additional Information

**How to cite this article**: Zhang, Q. *et al.* *Tcstv1* and *Tcstv3* elongate telomeres of mouse ES cells. *Sci. Rep.* **6**, 19852; doi: 10.1038/srep19852 (2016).

## Supplementary Material

Supporting figures 1-8 and tables 1-5
