# Supplementary material for: Tcstv1 and Tcstv3 elongate telomeres of mouse ES cells
Source: Sci Rep. 2016 Jan 27;6:19852. doi: 10.1038/srep19852 (PMC4728397; doi:10.1038/srep19852)
Supplement: Supporting figures 1-8 and tables 1-5 [file srep19852-s1.doc]

**Supplementary Information**

***Tcstv1* and *Tcstv3* elongate telomeres of mouse ES cells**

Qian Zhang, Jiameng Dan, Hua Wang, Renpeng Guo, Jian Mao, Haifeng Fu, Xiawei Wei and Lin Liu^*^

**Supplementary Figures 1-8**

**Supplementary Tables 1-5**

**
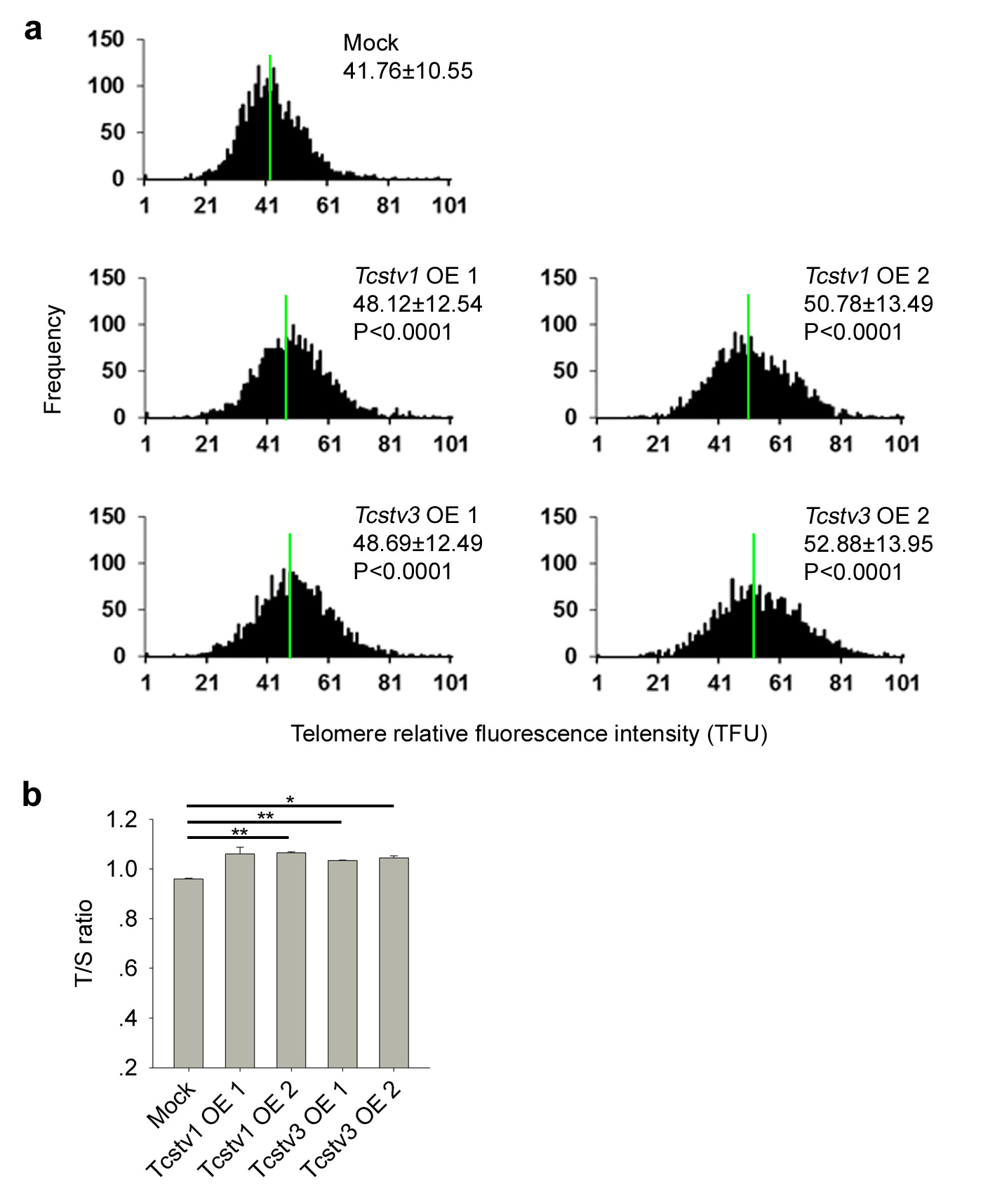
**

**Supplementary Figure 1.** Relative telomere lengths of *Tcstv1* OE, *Tcstv3* OE and mock ESCs at P15. **a**, Histogram shows distribution of relative telomere length expressed as fluorescence intensity (TFU, telomere fluorescence unit) by telomere Q-FISH analysis. Green line is median telomere length. Average telomere length is shown as mean TFU±SD. P value, compared to mock ESCs. **b**, qPCR analysis of telomere length shown as T/S ratio in *Tcstv1* OE and *Tcstv3* OE ESCs compared to mock ESCs. (*P<0.05; **P<0.01; two repeats with duplicates).

**
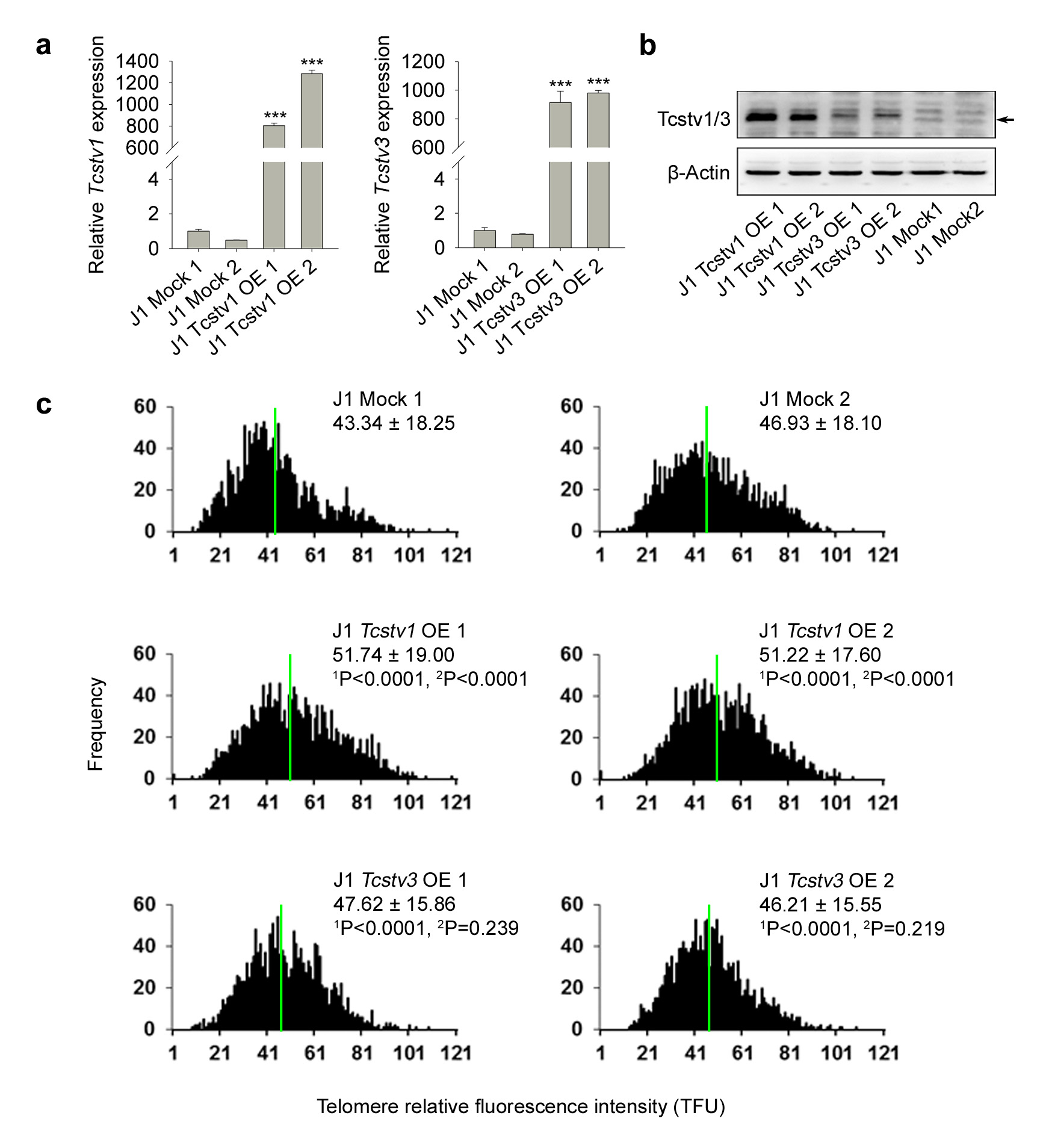
**

**Supplementary Figure 2.** Overexpression of *Tcstv1* or *Tcstv3* elongates telomere lengths in J1 ESC lines. **a-b**, Confirmation of overexpression of *Tcstv1* and *Tcstv3* in respective OE ESCs by qPCR (**a**; ***P<0.001, compared to both mock-1 and mock-2 ESCs; two repeats with duplicates) and western blot (**b**; Tcstv1/3 protein bands pointed by black arrow). **c**, Histogram shows distribution of relative telomere length expressed as fluorescence intensity (TFU, telomere fluorescence unit) by telomere Q-FISH analysis at P7. Average telomere length is shown as mean TFU±SD. ^1^P refers to P value compared to J1 mock-1 ESCs, and ^2^P refers to P value compared to J1 mock-2 ESCs by t-test.


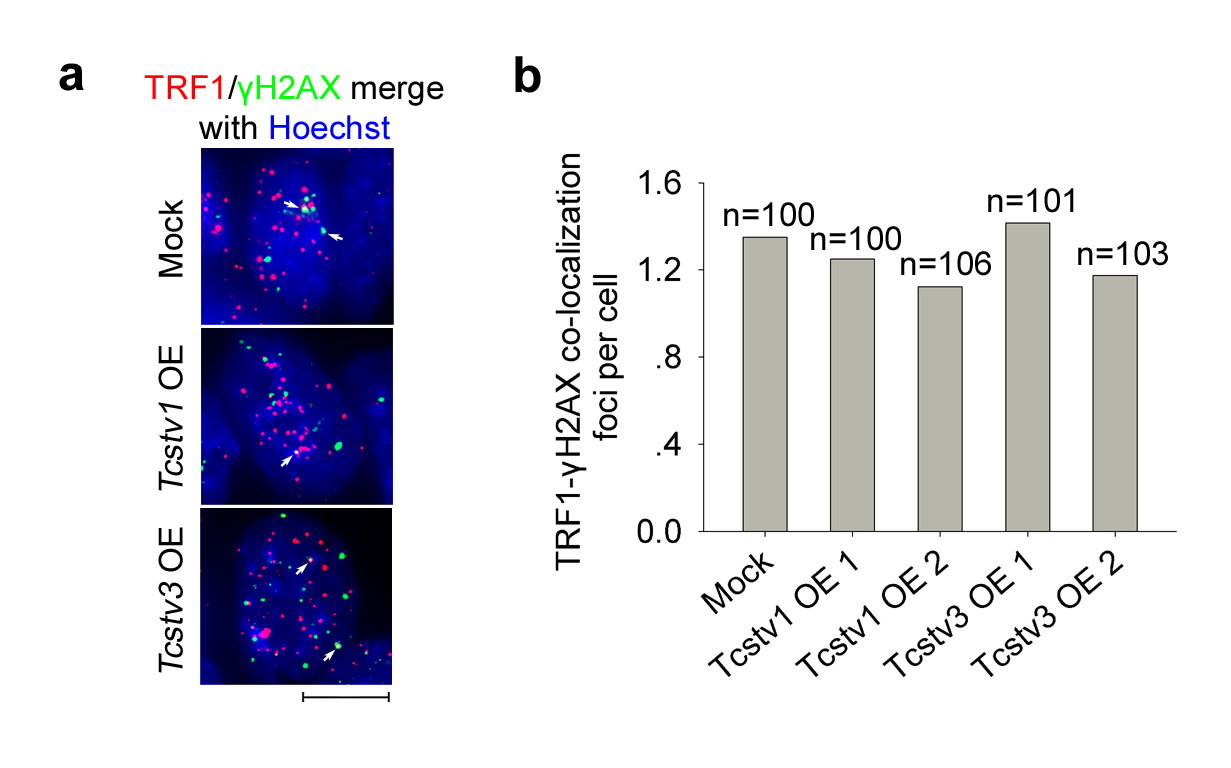


**Supplementary Figure 3.** Overexpression of *Tcstv1* or *Tcstv3* does not affect DNA damage at telomeres in ESCs. **a**, Immunofluorescence staining of γH2AX (green) at telomeres (TRF1, red) in *Tcstv1* OE, *Tcstv3* OE and mock ESCs. All nuclei were stained with Hoechst 33342 (blue). Yellow foci in the merged images indicate TRF1-γH2AX co-localization. Scale bar =10 μm. **b**, TRF1-γH2AX co-localization foci per cell in *Tcstv1* OE, *Tcstv3* OE and mock ESCs. n, number of cells counted. χ^2^ test and no statistical differences (P>0.05).


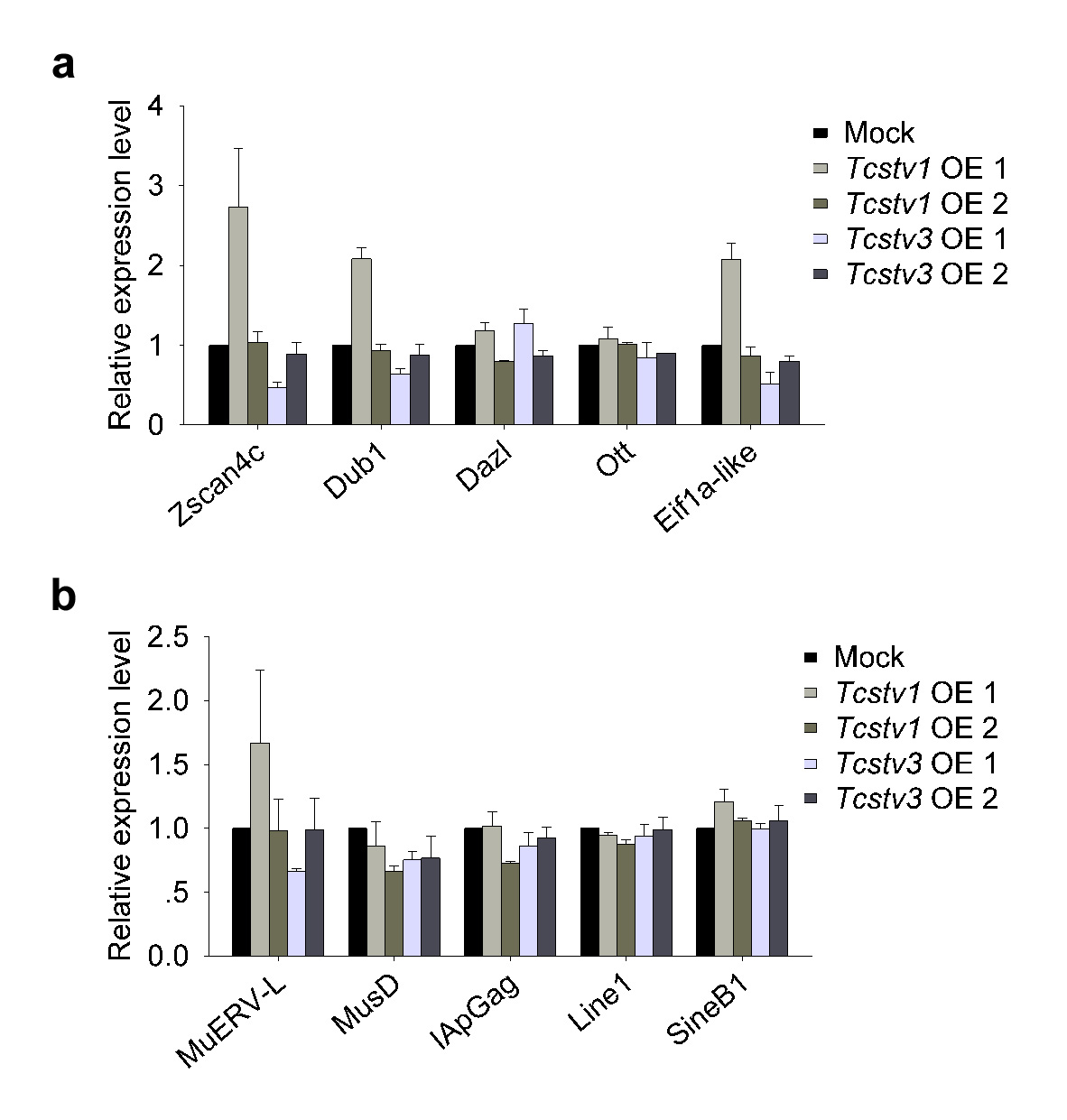


**Supplementary Figure 4.** Relative expression levels of genes expressed in 2C-state of ESCs and retrotransposons in *Tcstv1* OE, *Tcstv3* OE and mock ESCs. **a**, Expression levels of genes expressed in 2C-state of ESCs by qPCR analysis (two repeats with duplicates). **b**, Expression levels of retrotransposon genes by qPCR analysis.


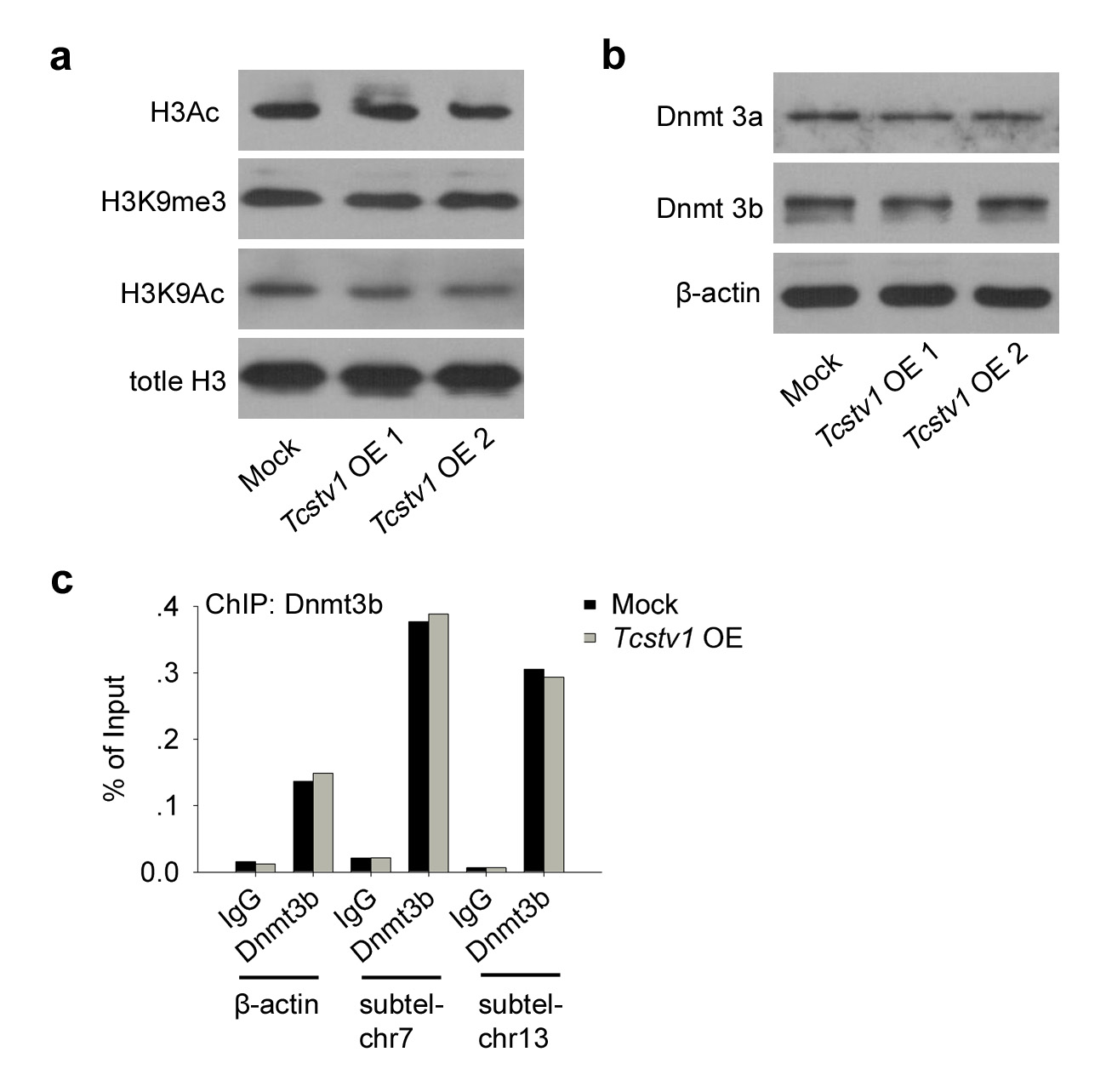


**Supplementary Figure 5.** Histone modification levels and expression of Dnmt3a, Dnmt3b protein following *Tcstv1* overexpression. **a**, Levels of histone H3Ac, H3K9me3 and H3K9Ac by western blot. **b**, Expression levels of Dnmt3a and Dnmt3b by western blot. **c**, ChIP-qPCR assay showing enrichment of Dnmt3b to subtelomeres following *Tcstv1* overexpression in ESCs.


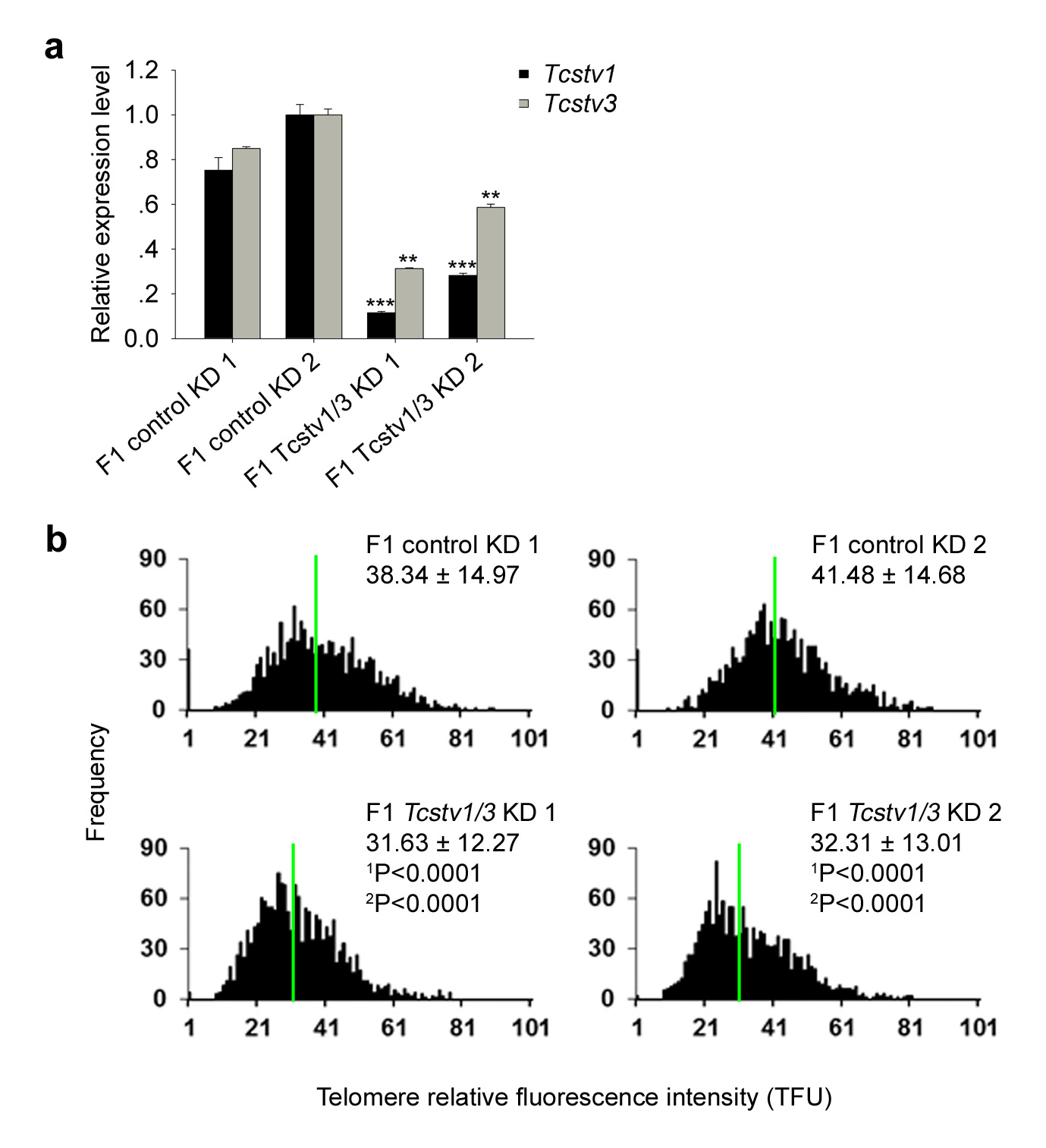


**Supplementary Figure 6.** *Tcstv1/3* knockdown shortens telomeres in F1 ESC lines. **a**, Confirmation of knockdown efﬁciency in *Tcstv1/3* KD ESCs generated by shRNA1 construct by qPCR. **P<0.01, ***P<0.001, compared to both control KD-1 and control KD-2 ESCs; two repeated experiments with duplicates. **b**, Histogram shows distribution of relative telomere length expressed as TFU (telomere fluorescence unit) by telomere Q-FISH analysis at P8. Average telomere length is shown as mean TFU±SD. ^1^P means P value compared to F1 control KD-1 ESCs, and ^2^P means P value compared to F1 control KD-2 ESCs by t-test.

**
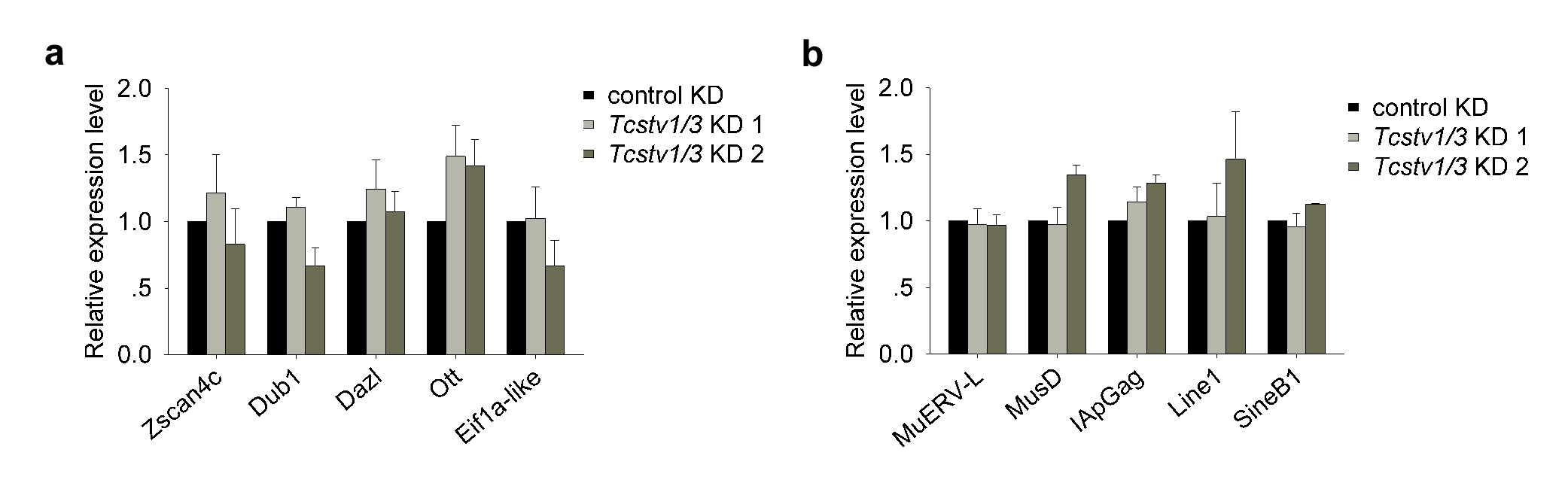
**

**Supplementary Figure 7.** Relative expression levels of genes expressed in 2C-state of ESCs and retrotransposons show no significant differences between *Tcstv1*/3 KD and control ESCs. **a**, Expression levels of genes expressed in 2C-state of ESCs by qPCR analysis (two repeats with duplicates). **b**, Expression levels of retrotransposon genes by qPCR analysis.

**
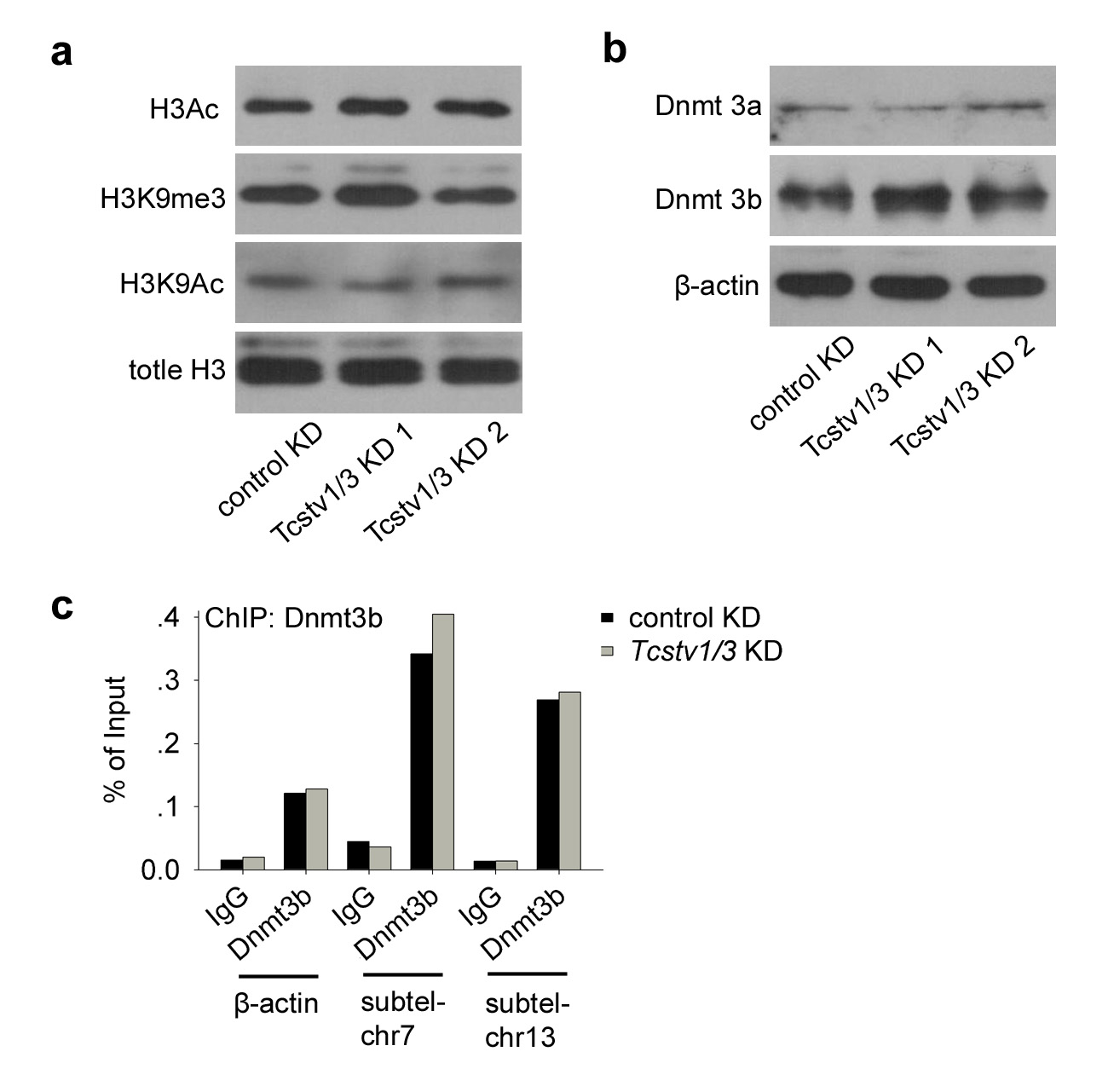
**

**Supplementary Figure 8.** *Tcstv1*/*3* knockdown does not alter histone modification levels, and expression of Dnmt3a and Dnmt3b. **a**, Levels of histone H3Ac, H3K9me3 and H3K9Ac by western blot. **b**, Expression levels of Dnmt3a, Dnmt3b protein by western blot. **c**, ChIP-qPCR assay showing that *Tcstv1/3* knockdown does not alter Dnmt3b binding to subtelomeres in ESCs.

**Supplementary Table 1.**

**Targeted genes by shRNA1 construct**

| **Gene bank accession number** | **Transcripts** |
| --- | --- |
| NM_018756 | Mus musculus 2-cell-stage, variable group, member 1 (Tcstv1), mRNA |
| NM_153523 | Mus musculus 2-cell-stage, variable group, member 3 (Tcstv3), mRNA |
| NM_199060 | Mus musculus cDNA sequence AF067061 (AF067061), mRNA |
| NM_001037925 | Mus musculus cDNA sequence BC147527 (BC147527), mRNA |
| NM_001039648 | Mus musculus predicted gene, 20767 (Gm20767), mRNA |
| NM_001033769 | Mus musculus RIKEN cDNA B020031M17 gene (B020031M17Rik), mRNA |
| XM_011244717 | PREDICTED: Mus musculus predicted gene, 21818 (Gm21818), mRNA |

**Supplementary Table 2.**

**19 nuclotide sequences for RNA knockdown of *Tcstv1/3***

| Control shRNA | GCGTTCAATTAGCAGACCA |
| --- | --- |
| shRNA1 | GGATACATCAGATATTGAA |
| shRNA2 | GGTGTTCTAATTCTGAGGC |

**Supplementary Table 3.**

**Primers used for quantitative real-time PCR analysis**

| **Gene name** | **Primer** | **Gene bank accession**  **number** |
| --- | --- | --- |
| GAPDH | TCAACAGCAACTCCCACTCTTCCA  ACCACCCTGTTGCTGTAGCCGTAT | NM_001289726 |
| Tcstv1 | TGAACCCTGATGCCTGCTAAGACT  AGATGGCTGCAAAGACACAACTGC | NM_018756 |
| Tcstv3 | AGAAAGGGCTGGAACTTGTGACCT  AAAGCTCTTTGAAGCCATGCCCAG | NM_153523 |
| Terc | CATTAGCTGTGGGTTCTGGTCT  TCCTGCGCTGACGTTTGTTT | NR_001579 |
| Tert | ACTGGTGGAGATCATCTTTCTGGG  ACCTGAGGAGTCTGACATATTGGC | NM_009354 |
| Oct4 | TTGGGCTAGAGAAGGATGTGGTT  GGAAAAGGGACTGAGTAGAGTGTGG | NM_013633 |
| Nanog | TTGCTTACAAGGGTCTGCTACT  ACTGGTAGAAGAATCAGGGCT | NM_028016 |
| Zscan4c | CCGGAGAAAGCAGTGAGGTGGA  CGAAAATGCTAACAGTTGAT | NM_001013765 |
| Dub1 | GCAGGCCAACCTCAAACAG  CGCAGGGCTCTCCTAAATCTT | NM_007887 |
| Dazl | ATGTCTGCCACAACTTCTGAG  CTGATTTCGGTTTCATCCATCCT | NM_010021 |
| Ott | ATGGCGAACCATGAAGACGAA  GTCCAGGGAAAAATGCTTGCC | NM_011022 |
| Eif1a-like  (Gm2022) | GGTAATGGAGTCCTGCTGTATTT  AGAGTCCTGGCTTCTGATAGT | NM_001177574 |
| MuERV-L | CCCATCATGAGCTGGGTACT  CGTGCAGATCCATCAGTAAA |  |
| MusD | GATTGGTGGAAGTTTAGCTAGCAT  TAGCATTCTCATAAGCCAATTGCAT |  |
| IAp-Gag | AATCTCAGAACCGCTCCATGA  TTTCTTAAAATGCCCAGGCTTT |  |
| Line1 | TTTGGGACACAATGAAAGCA  CTGCCGTCTACTCCTCTTGG |  |
| SineB1 | GTGGCGCACGCCTTTAATC  GACAGGGTTTCTCTGTGTAG |  |

**Supplementary Table 4.**

**Primers for qPCR analysis of telomere length**

| **Gene name** | **Primer** |
| --- | --- |
| mTel-F | CGGTTTGTTTGGGTTTGGGTTTGGGTTTGGGTTTGGGTT |
| mTel-R | GGCTTGCCTTACCCTTACCCTTACCCTTACCCTTACCCT |
| 36B4-F | ACTGGTCTAGGACCCGAGAAG |
| 36B4-R | TCAATGGTGCCTCTGGAGATT |

**Supplementary Table 5.**

**ChIP-qPCR primers for subtelomeres**

| **Gene name** | **Primer** |
| --- | --- |
| Subtel-chr7 | GCTGGACAGGTCAAGGTGGG  TGTAACGGCGAATGTGAGGG |
| Subtel-chr13 | TAAAGGCGTGAGCCACCACACTTA  TATGAGAGAGCAAGCAGGCCAAGT |
| β-actin | CGTGTGACAAAGCTAATGAGGCTG  CTAAGTTCAGTGTGCTGGGAGTCT |
